# Self-administered Web-Based Tests of Executive Functioning and Perceptual Speed: Measurement Development Study With a Large Probability-Based Survey Panel

**DOI:** 10.2196/34347

**Published:** 2022-05-09

**Authors:** Ying Liu, Stefan Schneider, Bart Orriens, Erik Meijer, Jill E Darling, Tania Gutsche, Margaret Gatz

**Affiliations:** 1 Center for Economic and Social Research University of Southern California Los Angeles, CA United States; 2 Leonard Davis School of Gerontology University of Southern California Los Angeles, CA United States; 3 Department of Psychology University of Southern California Los Angeles, CA United States

**Keywords:** cognitive tests, internet, probability-based, web-based, executive function, response speed, self-administered test, mobile phone

## Abstract

**Background:**

Cognitive testing in large population surveys is frequently used to describe cognitive aging and determine the incidence rates, risk factors, and long-term trajectories of the development of cognitive impairment. As these surveys are increasingly administered on internet-based platforms, web-based and self-administered cognitive testing calls for close investigation.

**Objective:**

Web-based, self-administered versions of 2 age-sensitive cognitive tests, the Stop and Go Switching Task for executive functioning and the Figure Identification test for perceptual speed, were developed and administered to adult participants in the Understanding America Study. We examined differences in cognitive test scores across internet device types and the extent to which the scores were associated with self-reported distractions in everyday environments in which the participants took the tests. In addition, national norms were provided for the US population.

**Methods:**

Data were collected from a probability-based internet panel representative of the US adult population—the Understanding America Study. Participants with access to both a keyboard- and mouse-based device and a touch screen–based device were asked to complete the cognitive tests twice in a randomized order across device types, whereas participants with access to only 1 type of device were asked to complete the tests twice on the same device. At the end of each test, the participants answered questions about interruptions and potential distractions that occurred during the test.

**Results:**

Of the 7410 (Stop and Go) and 7216 (Figure Identification) participants who completed the device ownership survey, 6129 (82.71% for Stop and Go) and 6717 (93.08% for Figure Identification) participants completed the first session and correctly responded to at least 70% of the trials. On average, the standardized differences across device types were small, with the absolute value of Cohen *d* ranging from 0.05 (for the switch score in Stop and Go and the Figure Identification score) to 0.13 (for the nonswitch score in Stop and Go). Poorer cognitive performance was moderately associated with older age (the absolute value of *r* ranged from 0.32 to 0.61), and this relationship was comparable across device types (the absolute value of Cohen *q* ranged from 0.01 to 0.17). Approximately 12.72% (779/6123 for Stop and Go) and 12.32% (828/6721 for Figure Identification) of participants were interrupted during the test. Interruptions predicted poorer cognitive performance (*P*<.01 for all scores). Specific distractions (eg, watching television and listening to music) were inconsistently related to cognitive performance. National norms, calculated as weighted average scores using sampling weights, suggested poorer cognitive performance as age increased.

**Conclusions:**

Cognitive scores assessed by self-administered web-based tests were sensitive to age differences in cognitive performance and were comparable across the keyboard- and touch screen–based internet devices. Distraction in everyday environments, especially when interrupted during the test, may result in a nontrivial bias in cognitive testing.

## Introduction

### Background

Internet-based surveys have received widespread attention as methods of large-scale data collection in many fields of health research. Although internet surveys have traditionally focused on the collection of self-reported data (eg, participants’ subjective attitudes, health behaviors, and self-reported medical conditions), interest in the ability to conduct objective testing of cognitive abilities over the internet has substantially increased in recent years [1-4]. Cognitive capacities are relevant to people’s ability to understand and act on information and serve as a basis for higher-order functioning and well-being. Large-scale monitoring of cognitive abilities is necessary to determine incidence rates, risk factors, and long-term trajectories of the development of cognitive impairment associated with chronic conditions [5] and normal cognitive aging [6].

Tests of cognitive functioning are often included in large population surveys administered face to face or over the telephone [7,8]. Compared with these conventional assessment strategies, large-scale internet-based cognitive testing has many potential benefits. Internet surveys have the obvious advantages of lower labor costs and quicker turnaround while achieving demographic representations of the population similar to those of traditional surveys [9]. Similar to conventional computerized testing where a psychometrist is present [10], web-based cognitive testing achieves higher precision and data quality because of electronic scoring compared with human-based scoring (eg, stopwatch), especially in timed assessments. Given that web-based cognitive tests are self-administered, do not require an examiner to be present, and eliminate manual entry of data, they can be much more flexibly and efficiently administered compared with conventional cognitive test formats [11]. Participants can be tested at various times on their own computers or mobile devices in their homes or in other daily environments; responses can be automatically routed and test responses stored electronically, thus making data available to researchers or other interested stakeholders in near real time. This allows for data collection with much larger and more diverse participant samples [3,12].

Notwithstanding the potential advantages of web-based cognitive testing, there are many open questions about the accuracy of its administration. Self-administered web-based cognitive tests reduce the level of standardization often seen in tests administered by trained professionals, which involves precisely controlled test environments and standardized equipment. The lack of standardization is particularly fraught for tests that are timed. Cognitive functioning scores may potentially differ markedly depending on participants’ computer skills and the used device [13,14], such as whether the test is taken on a laptop computer with a physical keyboard versus a tablet or smartphone without a keyboard [1,15]. Moreover, although self-administered web-based testing increases flexibility and convenience of test administration in participants’ daily lives, this comes with the potential costs of excessive measurement errors and biases associated with environmental influences (eg, the location where the test is taken and interruption by people or devices). To mitigate potential biases, it has been recommended that web-based cognitive tests should be specifically designed for unmonitored settings with clear instructions and consistent administration across operating systems, browsers, and devices [16]. Furthermore, as normative data may not be comparable across different modes of administration, norms should be made accessible specifically for web-based cognitive testing and generated from large and representative samples [13]. To date, available web-based cognitive tests have been predominantly developed for detecting age-related cognitive impairments in small and selected samples and lack normative information that could be applied in population-based studies [1,3].

To facilitate large-scale cognitive testing in the digital era, we developed web-based self-administered tests of perceptual speed and executive functioning in an existing US representative internet survey panel—the Understanding America Study (UAS). Panelists in the UAS complete monthly surveys and assessments on their own devices in everyday environments, providing an opportunity to evaluate the performance of these measures in a large and diverse sample. This allowed us to explore the extent to which the use of different web-based devices and environmental influences (eg, location or distractions) affects the precision of cognitive test scores and develop nationally representative norms for use in population-based research. Many web-based surveys and panels are opt-in samples recruited on the web, such as through Facebook advertisements. Using such a sample would potentially risk overrepresenting individuals with stronger connections to digital technology who are younger and have a higher socioeconomic status. To avoid such biases, the UAS recruits panel members using traditional methods (sampling addresses from the United States Postal Service Delivery Sequence File and initially contacting sampled individuals by postcards and letters) and provides them with a tablet and internet connection if they do not have access to the internet.

We focus on 2 cognitive domains—perceptual speed and executive functioning—that involve the speed of answering questions. Perceptual speed refers to how long an individual requires to take in information, understand it, and begin to act on it. It is typically measured as the time required to perceive and respond to visual information. Executive functioning entails the cognitive skills used to control behavior. Key executive functions include response inhibition (resisting automatic impulses to act), interference control (suppressing attention to unwanted information), and mental set shifting (being able to change perspectives) [17]. Both perceptual speed and executive functioning are fundamental cognitive skills that are distinct from other cognitive domains such as verbal, spatial, and memory abilities in that they are more sensitive to stress, depression, lack of sleep, and poor physical health [17-19]. Thus, they can be important mediators in health-related studies [20]. Although performance can be improved with training and practice [17,21], these skills decline with age [22] and serve as critical indicators of healthy aging or potential flags of impairment. Declines in perceptual speed have also been shown to portend other cognitive changes in older adults [23]. Furthermore, their time-dependent nature and proneness to environmental influences make them sensitive to errors or biases potentially induced by platform differences or everyday environments.

### Objectives of This Study

Our study had 3 objectives. The first was to examine differences in cognitive test scores across different web-based devices and the relationships between cognitive test scores and participant age. To do so, we asked the UAS panelists to complete the cognitive tests both on a keyboard-based computer (desktop or laptop) and on a device with a touch screen (tablet or smartphone) in a randomized order if both device types were available or complete the same test twice if only one type of device was available. The second objective was to study the extent to which test scores are related to environmental influences in everyday life, such as the location at which the test occurs and momentary distractions. The third objective was to provide test score norms based on a nationally representative sample in which individuals with cognitive impairment or potential dysfunction were not screened in or out. It is worth noting that there is no clinical gold standard; thus, these norms are not intended for clinical purposes, and thus, cognitive impairment cutoffs are outside the scope of our work.

## Methods

### Participants

The data for this study were collected as part of the UAS [24], a probability-based internet panel maintained at the University of Southern California [25]. In contrast to convenience (*opt-in*) panels, where participants self-select as members, UAS panelists are recruited through nationwide address-based sampling. This recruitment strategy relies on samples drawn with a known probability of selection from a US Postal Service list of all households in the nation, which tends to overcome many biases in population parameters estimated from convenience panels [26,27]. Participants without prior internet access are equipped with broadband internet and a tablet, which is important, given that internet access tends to be lower among older Americans and those with lower education [28]. UAS members are asked to complete 1 to 2 web-based assessments per month on various topics, including psychosocial well-being, economic concerns, retirement planning, decision-making strategies, and cognitive assessments. All active members of the UAS internet panel were asked to participate in these assessments, and no specific inclusion or exclusion criteria were applied for participation in this study.

The participants provided electronic informed consent for participation.

### Measures

#### Executive Functioning

We selected the Stop and Go Switching Task (SGST) developed for telephone administration by Lachman et al [7,29], which was implemented in the Midlife in the United States national longitudinal study. In the original phone-administered version, the experimenter says the word *red* or *green,* and the participant responds by saying either *stop* or *go*. The SGST comprises several conditions that are administered in series. In the normal condition, the participant responds *stop* to *red* and *go* to *green*. It is followed by a reverse condition where the participant responds *go* to *red* and *stop* to *green*. These 2 *baseline* conditions are followed by a mixed condition in which participants switch back and forth between normal and reverse instructions. The switch trials are the first response after the participant has to change from one condition to another. Nonswitch trials are those that do not involve a change in instructions. The participants practice each condition before beginning. Then, latencies are measured (based on audio recordings of the telephone assessments) between the cue and the response for the normal, reverse, switch, and nonswitch trials. The median response time in each type of trial is used as a score for one’s cognitive ability. The baseline normal condition measures choice reaction time, the reverse condition requires response inhibition, and the mixed condition requires task switching all of which are aspects of executive functioning [7].

#### Perceptual Speed

The test of perceptual speed that we selected was the Figure Identification test, which was originally developed as a paper-and-pencil test. This is based on the work of Thurstone [30] on primary mental abilities. The participant sees a target figure on top of 5 horizontally aligned similar figures. All figures are in black and white and vary in complexity, with some but not all representing recognizable objects (eg, an abstract dog or boat). The task is to identify 1 figure among the 5 that exactly matches the target as quickly as possible while being accurate. Perceptual speed is measured by counting the number of figures correctly circled on paper within a preset time limit. The paper-and-pencil version of the Figure Identification test has long been used as part of the Dureman and Sälde battery [31], especially in studies of cognitive aging [32].

### Web-Based Adaptation of the Cognitive Tests

#### Prior Adaptation for the Web

Developers had already taken steps to develop web-based versions of both cognitive tests. As much as possible, they emulated the original tests, except that the stimuli were presented on an electronic visual display (rather than on paper or via telephone) and responded to by pressing keys on a keyboard or buttons on a touch screen (rather than circling responses on paper or responding verbally), and responses and reaction times were electronically captured. For the Figure Identification test, respondents pressed the correct answer rather than circling the correct answer on a sheet of paper showing the figures (personal communication, Johansson). For SGST, participants view the word *red* or *green* and respond by pressing the *S* (stop) or *G* (go) key rather than answering verbally. Viewing words rather than viewing, for example, a red or green disk, eliminates the issues of color blindness. To minimize the motor component in response time, participants were encouraged to keep their fingers on the keys (JJ McArdle, CA Prescott, EE Walters, GG Fisher, B Helppie McFall, K Peters, unpublished user documentation, May 15, 2018). Administration of the version, as developed by JJ McArdle, CA Prescott, EE Walters, GG Fisher, B Helppie McFall, and K Peters (unpublished user documentation, 2018), to a sample of 408 participants who completed the SGST both by phone and web found longer response times for web than for phone for normal and reverse baseline conditions, longer response times for phone than for web for switch trials, and no difference for nonswitch trials (R McCammon, personal communication, January 11, 2022).

#### Further Adaptation in This Study

The UAS team administers surveys using the NubiS data collection tool, an open-source, secure data collection, storage, and dissemination system [33] developed at the Center for Economic and Social Research, University of Southern California. Surveys in NubiS are conducted in a web browser environment designed to optimize the harmonization of the survey experience across a wide variety of devices and browsers. This avoids the need to accommodate changes in the device or the web browser environment. Given the specific user interaction mechanisms of the web-based cognitive tests, the surveys were further refined to be administered on both devices with a keyboard and mouse (eg, desktop or laptop computers) and touch screen-based devices (eg, tablets or smartphones). For keyboard-based devices, the interface responds to keys pressed on the keyboard or mouse clicks. For touch screen–based devices, the interface incorporates buttons for the possible answer keys, which, when pressed, simulate the behavior of their respective keyboard or mouse counterparts.

**Figure 1:**
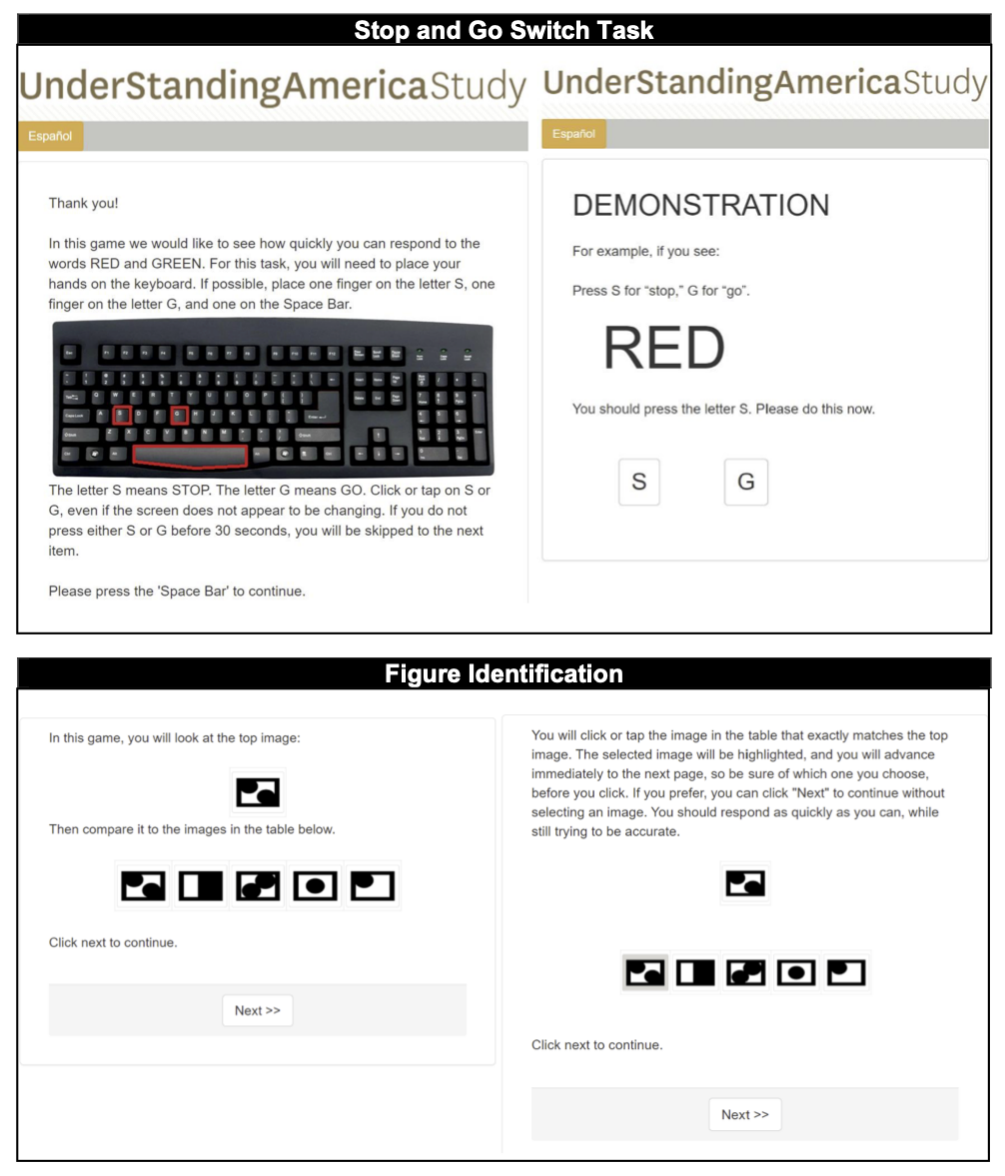
Screenshots of the introductory (left) and demonstration (right) pages of web-based versions of the Stop and Go Switching Task (top) and the Figure Identification test (bottom).

Figure 1 presents selected screenshots of the web-based versions. At the beginning of each test, participants were given a brief introduction to the task (see the left panels in Figure 1), followed by a demonstration (right panels). After the demonstration, practice trials were provided with automated feedback before participants were asked to start the test. In the feedback, participants were told whether their answer was correct or incorrect; if incorrect, they were shown the correct answer and were given another practice trial. The participants were also instructed to set aside the uninterrupted time to complete the tasks.

For both tests, item latencies were recorded in the client browser as time lapsed, in milliseconds, between the moment of *screen fully loaded* to the moment that an answer key was pressed or the button was clicked (captured through a JavaScript *onkeyup* event). The event of a fully loaded screen or page is captured by a JavaScript document-ready expression from the client’s browser. In this way, browser speed differences displaying the page are excluded from the latency data, as is the time spent on the server-client interaction. This ensures that any differences in platform or browser speed in displaying or internet speed do not affect the recorded response latencies.

### Pilot Testing of the Web-Based Versions

A combined total of 964 UAS panelists were initially recruited for a sequence of pilot tests and to provide feedback about their experiences while performing the web-based tests. Feedback was provided by respondents at the end of the pilot surveys via closed-ended questions about the usability of various devices, clarity of instructions, and an open-ended text box for additional comments. The authors (JD, SS, YL, EM, and MG) used descriptive statistics to examine responses to the feedback questions and indicators such as item and survey nonresponse, break off, and level of compliance with written directions. Several refinements were made during the iterative process, informed by the pilot data and participant feedback. First, the number of practice sessions for the SGST was increased from 1 to 3 practice sessions for the baseline and reverse conditions after 2 initial demonstrations to ensure that all participants were fully aware of the specifics of the test for all trials. Second, the wording of the instructions was refined to increase clarity in response to participant feedback. Third, we simplified the presentation and layout on the screen by eliminating superfluous elements (eg, a *refuse* button) on the screen. We also minimized the number of different keys and streamlined the number of hand motions that the participants needed to use throughout the tests. For example, in the SGST, when a respondent is asked to keep their fingers on certain keys on the keyboard, using the spacebar to move to the next page is more natural than pressing the *next* button, which requires operating a mouse (see the top left panel in Figure 1).

### Main Study Procedures and Measures

#### Overview

To compare the test performance when using a device with a keyboard and mouse versus a touch screen, we designed a study in which participants were asked to complete the tests twice in separate modules. Those who had access to both device types were randomized into using a keyboard and mouse first and touch screen second, or vice versa, whereas participants who had access to only 1 of the 2 types were asked to complete the tests twice on the same device. Leveraging the within-person design resulted in a much reduced and nonrepresentative sample (1770 instead of 6129 for SGST and 1892 instead of 6717 for the Figure Identification test). Therefore, our primary analyses were based on the participants’ first completed test (ie, based on a between-person comparison across device types). However, we also report results from sensitivity analyses conducted in a reduced sample of participants who provided data for the within-person comparison across device types, which is in line with the original study design.

The types of devices used by participants were monitored using Mobile Detect, a hypertext preprocessor–based tool for analyzing and classifying browser user agent strings [34]. When entering each test session, the tool automatically analyzed the browser user agent string to determine the device type, and the survey instructed the participants to confirm or switch devices if the detected type was inconsistent with the assigned type. The SGST and Figure Identification tests were administered separately, which were an average of 10.6 (SD 53.1) days apart.

#### SGST Scoring

The SGST included 10 normal and 10 reverse baseline trials, as well as 23 nonswitch and 6 switch trials. Following prior research using the telephone-administered version [7], each of these 4 types of tasks was scored using median response times across all trials within each trial type to reduce the effects of outlier responses. Participants who failed to meet an acceptable level of overall accuracy (at least 70% correct trials) were not scored. Higher scores indicate slower median response times and, thus, poorer executive functioning. The test items were simple enough that anyone could answer them correctly when spending sufficient effort. The response times then measured the concepts of interest as the amount of effort required to respond. When a respondent answers many items incorrectly, it, therefore, likely indicates problems such as carelessness or inattention, which may correspond to rushing through the items, making the response times less valid measures of the concepts we intended to measure. To guard against this, we followed the literature and excluded respondents with too many incorrect items. There is a trade-off between validity at the individual level and representativeness and the overall sample size. We considered several cutoffs and, based on descriptive analyses of early data extracts, concluded that 70% best balanced the 2 objectives in this trade-off.

#### Figure Identification Test Scoring

In the original paper-and-pencil version, the Figure Identification test was administered in 2 sets of 30 items each. Participants were given 120 seconds per set to complete as many items as they could, and the final score was the total number of figures that a person had solved correctly within the preset time limits [31]. In this study, administering the test on the web made it unnecessary to terminate the test after a preset time limit, as we were able to set posterior time limits [35]. Therefore, we asked participants to complete all 60 figures. To eliminate potential order effects, the figures were grouped into 6 blocks of 10, and the order of the blocks was randomized. We used the median and IQR of the item-level response times obtained from the pilot data as proxies of item difficulty and discrimination measures when creating the blocks of items. The 6 items with the lowest median were randomly assigned to the 6 blocks, and so were the remaining items. To ensure the resulted blocks were comparable, we repeated this process 500 times and chose a combination with a similar overall median and IQR across blocks.

To derive a final Figure Identification test score, we used a posterior time limit of 90 seconds per 30-item set as preliminary analyses indicated ceiling effects when the original 120-second limit was used, with many participants obtaining scores approaching the maximum possible score. It is well-known that electronic responses tend to be faster than paper-based because of differences in item presentation and response format (eg, pressing a key instead of marking a mark with a pen) [36]. Thus, the final score was the total number of correctly identified figures within 2×90 seconds. As with the SGST, we required that a respondent had at least 70% correct figures to be scored. The rationale was to screen out inattentive responders as our prior research has shown that having <70% figures incorrect on the Figure Identification test is an indicator of careless, inattentive responding and likely yields invalid test results [37]. Extreme outlier response times >30 seconds (99.80th percentile across all responses) were removed from the data and did not count toward the respondent’s correctly solved figures. Higher scores on the test reflect a faster perceptual speed.

Although, in combination, the scoring of the Figure Identification test reflects both the speed and accuracy of responses, it is also of interest to examine these components separately. To date, it is not known whether device differences or environmental influences in self-administered web-based cognitive tests affect response speed, response accuracy, neither, or both. Therefore, as a secondary set of scores derived from the test, we also examined (1) response inaccuracy (ie, the percentage of figures a respondent had incorrect) and (2) participants’ response latency (ie, the median response time of all accurate trials) as separate outcome measures. For both measures, higher scores reflected worse performance.

#### Posttest Survey on Readability and Screen Navigation

After each test session, the participants were asked about their experiences. This included (1) how clearly they were able to see the text, buttons, or figures (very clearly, not very clearly, and not clearly at all); (2) difficulty navigating the screen such as tapping, clicking, or needing to scroll (very easy, somewhat easy, somewhat difficult, and very difficult); and (3) the overall experience (eg, needing to scroll or move the screen to see all content, content obscured by something else, or no technical difficulties). They were also provided an open-ended text box to leave additional comments.

#### Environmental Influences

Information on participants’ current location and momentary distractions was collected via self-report immediately after each session of the cognitive test. A single item asked the participants where they were when they completed the test (at home or their residence, at work, at school, in a public place, riding a car or other transportation, or walking outside), which was recoded as being at home (1) or not (0). To assess momentary distractions, participants were asked about other activities they performed while they completed the test (talking to other people, listening to music or podcasts, watching television, playing games, following content on the internet, texting, or checking their email). Each activity option was coded as yes (1) or no (0). Finally, participants were asked whether they were interrupted by anything while completing the test (answers: yes or no).

#### Demographic Variables

Demographic information was collected quarterly from the UAS survey panel. This included sex, age, race and ethnicity, education, and household income. The mean age of participants in this study was 49.6 (SD 15.8, range 18-101) years for the analysis of the SGST and 50.5 (SD 16.0, range 18-102) years for the Figure Identification test. Approximately three-fifths were female (3620/6129, 59.06% for SGST; 4021/6717, 59.86% for Figure Identification), approximately two-thirds were White (4030/6119, 65.86% for SGST; 4323/6708, 64.45% for Figure Identification), slightly less than half had a bachelor’s degree or more (2732/6127, 44.59% for SGST; 2797/6715, 41.65% for Figure Identification), and approximately three-fifths had a household income of ≥US $50,000 (3730/6113, 61.01% for SGST; 3922/6701, 58.53% for Figure Identification).

### Analysis Strategy

The analyses were conducted separately for the collected SGST and Figure Identification test data. For primary analyses of each cognitive test, we included all participants who completed at least one session of the test and responded correctly to at least 70% of the trials, regardless of the type of device used. Sensitivity analyses examined within-person differences in test scores among the smaller group that completed the tests on both device types. Demographic characteristics were compared across device ownership groups using the Pearson chi-square test. A comparison of the cognitive scores across the keyboard- and touch screen–based devices was performed in 2 ways. First, we used Cohen *d* as a measure of the standardized mean difference. We considered the standardized mean difference to be small with *d*=0.20, medium with *d*=0.50, and large with *d*=0.80 [38]. Second, Pearson correlation was used to quantify the association between the cognitive scores and the respondent’s age, where scatterplots were inspected to detect potential nonlinear relationships. Cohen *q* was used to quantify differences in the age correlation across device types, where values of 0.10, 0.30, and 0.50 can be interpreted as small, medium, and large effects, respectively [38]. To understand the association between cognitive scores and environmental influences, multivariable regression was used with age as a covariate. We controlled for age in this analysis to avoid omitted variable bias, given that cognitive performance is hypothesized to decline as one’s age increases, and environmental influences may also be related to age. Effects at *P*<.05 were considered statistically significant. Finally, US population norms for cognitive scores were computed as weighted mean cognitive scores for different age groups using standard UAS survey weights [39]. All analyses were performed using Stata (version 16) and SAS (version 9.4).

### Ethics Approval

This study was approved by the institutional review board of the University of Southern California (UPS 14-00148).

## Results

### Descriptive Characteristics of the Analysis Sample

For the SGST, among the 9453 panelists invited to participate, 7410 (78.39%) completed the device ownership survey. Of those 7410 individuals, 7039 (94.99%) completed the first session, and 6129 (82.71%) correctly responded to at least 70% of the trials and received a score; one case was excluded from the analysis because of a lack of information about the device they used. For the Figure Identification test, 9445 were invited, and 7216 (76.4%) participants completed the device ownership survey. Of those 7216 individuals, 6879 (95.33%) completed the first session, and 6717 (93.08%) correctly identified at least 70% of the figures and were analyzed. A small proportion of the participants (194/7292, 2.66% for SGST; 9/6888, 0.13% for the Figure Identification test) dropped out after the practice trials.

Table 1 shows the demographic composition of the analytic samples overall and by the devices owned. Those with only a touch screen device were younger, had lower education, had lower household income, and were less likely to be male or White than those with only a keyboard device. Individuals who had both types of devices tended to have higher education and household income and were slightly younger and more likely to be White than single-device owners.

**Table 1:** Demographic characteristics of the study samples.

Participant characteristics			Stop and Go Switching Task (n=6129)						*P* value			Figure Identification test (n=6717)						*P* value
			Both devices, n (%)		Keyboard only, n (%)		Mobile only, n (%)				Both devices, n (%)			Keyboard only, n (%)		Mobile only, n (%)		
			(n=3228)		(n=959)		(n=1942)				(n=3345)			(n=1172)		(n=2200)		
**Age (years)**									<.001									<.001
	18-34	687 (21.28)		99 (10.3)		443 (22.81)				684 (20.45)			100 (8.53)		475 (21.59)			
	35-44	688 (21.31)		129 (13.5)		474 (24.41)				703 (21.02)			156 (13.31)		520 (23.64)			
	45-54	623 (19.3)		154 (16.1)		365 (18.8)				635 (18.98)			165 (14.08)		433 (19.68)			
	55-64	591 (18.31)		226 (23.6)		368 (18.95)				643 (19.22)			280 (23.89)		408 (18.55)			
	65-74	501 (15.52)		239 (25)		225 (11.59)				516 (15.43)			293 (25)		287 (13.05)			
	≥75	134 (4.15)		110 (11.5)		67 (3.45)				164 (4.9)			178 (15.19)		77 (3.5)			
**Race**									<.001									<.001
	Non-Hispanic White	2155 (66.76)		692 (72.3)		1183 (60.92)				2184 (65.29)			850 (72.53)		1289 (58.59)			
	Non-Hispanic Black	210 (6.51)		46 (4.8)		167 (8.6)				237 (7.09)			72 (6.14)		204 (9.27)			
	Hispanic	490 (15.18)		115 (12)		422 (21.73)				519 (15.52)			130 (11.09)		514 (23.36)			
	Non-Hispanic other	367 (11.37)		104 (10.9)		168 (8.65)				400 (11.96)			119 (10.15)		190 (8.64)			
**Sex**									<.001									<.001
	Men	1332 (41.26)		482 (50.3)		695 (35.79)				1342 (40.12)			604 (51.54)		750 (34.09)			
	Women	1896 (58.73)		477 (49.7)		1247 (64.21)				2003 (59.88)			568 (48.46)		1450 (65.91)			
**Education**									<.001									<.001
	High school or less	433 (13.41)		203 (21.2)		592 (30.48)				478 (14.29)			267 (22.78)		724 (32.91)			
	Some college	1028 (31.85)		322 (33.6)		817 (42.07)				1113 (33.27)			415 (35.41)		921 (41.86)			
	Bachelor or more	1766 (54.71)		434 (45.3)		532 (27.39)				1754 (52.44)			490 (41.81)		553 (25.14)			
**Household income (US $)**									<.001									<.001
	≤24,999	396 (12.27)		159 (16.6)		541 (27.86)				453 (13.54)			216 (18.43)		671 (30.5)			
	25,000-49,999	573 (17.75)		233 (24.4)		481 (24.77)				598 (17.88)			283 (24.15)		558 (25.36)			
	50,000-99,999	1139 (35.29)		343 (35.9)		537 (27.65)				1167 (34.89)			420 (35.84)		579 (26.32)			
	≥100,000	1109 (34.36)		221 (23.1)		381 (19.62)				1119 (33.45)			247 (21.08)		390 (17.73)			

### Readability and Test Experience

Of the 6129 and 6717 participants who met the 70% correctness threshold to be in the analysis samples for the SGST and the Figure Identification tests, 5901 of 6129 (96.28 for SGST) and 6492 of 6715 (96.68% for the Figure Identification test) reported seeing the text and buttons very clearly, 5746 of 6129 (93.75% for SGST) and 6372 of 6714 (94.91% for the Figure Identification test) considered it very or somewhat easy to navigate the screen, and 5231 of 6129 (85.35% for SGST) and 6075 of 6710 (90.54% for the Figure Identification test) reported that the text and buttons fit on the same screen with no technical difficulties, respectively. Those who completed the session but did not meet the 70% correctness threshold reported somewhat more problems. For the SGST, 73.7% (671/910) reported seeing the content very clearly, 72.7% (662/910) considered it very or somewhat easy to navigate, and 66% (598/906) had no technical difficulties; for the Figure Identification test, 63.8% (88/138) reported seeing the content very clearly, 70.8% (97/137) considered it very or somewhat easy to navigate, and 54.8% (74/135) had no technical difficulties.

### Comparison of Cognitive Scores Across Device Types

The results of the primary analyses of SGST are shown in the top panel of Table 2. The average SGST scores, which are the median response times for each type of trial, ranged from 0.92 to 1.53 seconds. The switch trials were the slowest, and the nonswitch trials were the fastest. The difference across device types was small on average, with scores on touch screen devices being 0.03 to 0.06 seconds slower than on keyboard devices. Standardized mean differences (Cohen *d*) ranged from 0.05 (for switch trials) to 0.13 (nonswitch trials), which is below the threshold of 0.20 for a small effect. All 4 scores were positively correlated with age (correlations ranged from 0.32 to 0.50), indicating a slower performance with higher age. The age correlations were similar across platforms; Cohen *q* for differences in age correlations ranged in absolute value from 0.01 (for switch trials) to 0.09 (for reverse baseline trials), which is below the threshold of 0.10 for a small effect.

**Table 2:** Comparison of cognitive scores and their correlation with age across device types.

Cognitive scores			Values, mean (SD)				Cohen *d* (95% CI)		Correlation with age				Cohen *q*
			Keyboard		Touch screen				Keyboard		Touch screen		
**Stop and Go Switching Task^a^**													
	Baseline^b^	1.04 (0.56)		1.09 (0.80)		0.07 (0.02 to 0.12)		0.38		0.32		0.07	
	Reverse baseline^b^	1.10 (0.58)		1.16 (0.59)		0.10 (0.05 to 0.15)		0.35		0.43		−0.09	
	Nonswitch^b^	0.92 (0.32)		0.97 (0.34)		0.13 (0.08 to 0.18)		0.48		0.50		−0.03	
	Switch^b^	1.50 (0.70)		1.53 (0.63)		0.05 (−0.01 to 0.10)		0.35		0.34		0.01	
**The Figure Identification test^c^**													
	The Figure Identification test score^d^	41.51 (8.43)		41.96 (8.36)		0.05 (0.01 to 0.10)		−0.61		−0.49		−0.17	
	Percentage figures incorrect^e^	5.70 (4.97)		7.40 (5.70)		0.32 (0.27 to 0.37)		−0.04		−0.05		0.00	
	Median response times^b^	4.76 (1.78)		4.53 (1.74)		−0.13 (−0.18 to −0.08)		0.56		0.45		0.15	

^a^For Stop and Go Switching Task, keyboard n=2820 and touch screen n=3309.

^b^Means and SDs are presented for seconds.

^c^For the Figure Identification test, keyboard n=3182 and touch screen n=3309.

^d^Means and SDs are presented for the number of figures.

^e^Means and SDs are presented for percentage.

As shown in the bottom panel of Table 2, the average Figure Identification test scores were very similar across device types, with a mean score of 41.5 (SD 8.43, possible range 0-60) for keyboard and 41.96 (SD 8.36) for touch screen devices (*d*=0.05). The scores showed pronounced negative correlations with age (*r*=−0.61 when completed on a keyboard device and *r*=−0.49 when completed on a touch screen device). The age relationship was stronger for the keyboard than for the touch screen (*q*=−0.17, just less than halfway between a small and medium effect size). The results for the secondary Figure Identification outcome measures suggested that participants made more mistakes when using a touch screen device (average percent incorrect figures 7.4, SD 5.70) than when using a keyboard device (average percent incorrect figures 5.7, SD 4.97; *d*=0.32, just less than half way between a small and medium effect), whereas response times were faster on average when using a touch screen device (mean of median response times 4.53, SD 1.74) than when using a keyboard device (mean of median response times 4.76, SD 1.78; *d*=0.13, a small effect). Older age was weakly associated with a lower percentage of incorrect figures (*r*=−0.044 for keyboard and *r*=−0.045 for touch screen; *q*=0.001) and strongly associated with slower responses (*r*=0.56 for keyboard and *r*=0.45 for touch screen; *q*=0.15). No meaningful nonlinear trend was observed between age and SGST or the Figure Identification test scores.

As a sensitivity check, we also conducted a *within-person* comparison of test scores among the smaller subgroup of participants who completed the cognitive tests consecutively on both device types. This analysis included 46.44% (1770/3811) of SGST participants and 55.76% (1892/3393) of Figure Identification test participants who owned both device types, took the test twice using a keyboard and touch screen device, and met the 70% correctness threshold for both sessions. Multimedia Appendix 1 Table S1 shows that respondents who met these criteria were younger on average; had higher education; had a higher household income; were more likely White; and (for the SGST sample) more likely male compared with respondents who owned both device types but did not meet the criteria. As shown in Multimedia Appendix 2 Table S2, respondents included in the within-person comparison showed somewhat faster response times on the SGST and better scores on the Figure Identification test (for both device types) compared with the full analysis sample. Within-person analyses of mean differences between devices largely replicated the results in the full sample, with effect sizes that were somewhat more pronounced for SGST and slightly smaller for the Figure Identification test. Correlations of the cognitive scores across device types were moderate to large, ranging from 0.37 (baseline trials in SGST) to 0.83 (median response times in the Figure Identification test).

### Environmental Influences

Participants were allowed to take the cognitive tests at the time and location of their preferences. Hence, although they were instructed to set aside some uninterrupted time before the tests, it is possible that the participants experienced distractions during testing. Using self-reports on environmental factors, Table 3 shows that approximately 89.6% (5486/6123 for the SGST sample) and 89.57% (6020/6721 for the Figure Identification test) of the participants took the tests at home. Approximately 12% to 13% of participants (779/6122, 12.72% for the SGST sample; 828/6718, 12.33% for the Figure Identification test) reported being interrupted while completing the test. Watching television (930/6101, 15.24% for the SGST sample; 962/6696, 14.37% for the Figure Identification test), listening to music or podcasts (518/6101, 8.49% for the SGST sample; 576/6696, 8.6% for the Figure Identification test), and talking with others (398/6101, 6.52% for the SGST sample; 483/6696, 7.21% for the Figure Identification test) were the most frequently reported distractors.

Tables 4 and 5 report the results of the regression analysis relating cognitive scores to potential distractions while controlling for age. Being interrupted during the SGST task was associated with significantly (*P*<.01) poorer performance on all subtests, with median response times being 0.07 (nonswitch trials) to 0.15 (switch trials) slower for participants who reported being interrupted. Watching television was predictive of slower responses on the nonswitch (0.03 seconds slower; *P*=.02) and switch trials (0.06 seconds; *P*=.007), and texting or checking email was predictive of slower responses on switch trials (0.23 seconds; *P*=.02). For the Figure Identification test, being interrupted during the task was associated with lower scores (0.89 fewer figures identified during the time limit; *P*=.002), as were watching television (1.44 fewer figures identified; *P*<.001), texting or checking email (2.87 fewer figures; *P*<.001), and playing another game during the task (4.21 fewer figures; *P*=.047). The results for the secondary Figure Identification test outcomes showed that watching television and following content on the internet were associated with a higher percentage of incorrect figures, whereas being interrupted, watching television, and texting or checking email were associated with slower median response times.

We also examined the association between environmental influences and age. If environments and distractions varied by age, this could at least partially account for the observed age differences in cognitive test scores. Younger age was significantly associated with a greater likelihood of taking the test away from home (*r*=.12, *P*<.001, both for the SGST and the Figure Identification test samples), being interrupted (*r*=.04, *P*<.001, SGST; *r*=.05, *P*<.001, Figure Identification test), talking (*r*=.09, *P*<.001, SGST; *r*=.12, *P*<.001, Figure Identification test), listening to music (*r*=.08, *P*<.001, SGST; *r*=.08, *P*<.001, Figure Identification test), following content on the internet (*r*=.05, *P*<.001, SGST; *r*=.04, *P*=.001, Figure Identification test), and texting or checking email during the test (*r*=.03, *P*=.008, Figure Identification test only). However, these factors did not meaningfully affect the relationship between age and cognitive scores. When comparing zero-order correlations between age and cognitive test scores with partial correlations that controlled for environmental influences, the correlations differed by less than *q*=0.01 for all tests.

**Table 3:** Frequency distribution of environmental influence factors.

Environmental influences	SGST^a^ (n=6123), n (%)	Figure Identification test (n=6721), n (%)
Interrupted during test	779 (12.72)	828 (12.32)
Being at home^b^	5486 (89.6)	6020 (89.57)
Watching television	930 (15.19)	962 (14.31)
Listening to music or podcast	518 (8.46)	576 (8.57)
Talking to others	398 (6.69)	483 (7.19)
Texting or checking email	42 (0.69)	45 (0.67)
Following content on internet	41 (0.67)	41 (0.61)
Playing another game	7 (0.11)	11 (0.16)

^a^SGST: Stop and Go Switching Task.

^b^Alternative responses to being at home included taking the test at work, at school, in a public place, riding a car or other transportation, or walking outside.

**Table 4:** Regression results associating environmental distractors with the Stop and Go Switching Task performance (N=6095).

Environmental influences	Normal baseline			Reverse baseline			Nonswitch			Switch	
	*b* (SE)	*P* value	*b* (SE)		*P* value	*b* (SE)		*P* value	*b* (SE)		*P* value
Interrupted during test	0.08 (0.03)	.006	0.08 (0.02)		<.001	0.07 (0.01)		<.001	0.15 (0.03)		<.001
Being at home	0.01 (0.03)	.81	0.01 (0.02)		.54	0.02 (0.01)		.13	0.01 (0.03)		.61
Watching television	0.02 (0.02)	.50	0.03 (0.02)		.11	0.03 (0.01)		.02	0.06 (0.02)		.007
Listening to music or podcasts	0.03 (0.03)	.33	0.01 (0.03)		.77	0.00 (0.01)		.84	−0.01 (0.03)		.80
Talking to others	−0.03 (0.04)	.49	0.03 (0.03)		.35	0.00 (0.02)		.99	0.03 (0.04)		.44
Texting or checking email	0.09 (0.11)	.42	0.13 (0.09)		.13	0.00 (0.05)		.93	0.23 (0.10)		.02
Following content on the internet	0.02 (0.11)	.86	0.08 (0.09)		.34	−0.01 (0.05)		.83	0.00 (0.10)		.97
Playing another game	0.26 (0.25)	.29	−0.01 (0.21)		.97	0.11 (0.11)		.34	0.41 (0.24)		.08

**Table 5:** Regression results associating environmental distractors with the Figure Identification test performance (N=6687).

Environmental influences	Figure Identification test score		Percentage incorrect			Median response time	
	*b* (SE)	*P* value	*b* (SE)	*P* value	*b* (SE)		*P* value
Interrupted during test	−0.89 (0.28)	.002	.0.31 (0.22)	.16	0.18 (0.06)		.003
Being at home	−0.18 (0.29)	.54	−0.14 (0.22)	.53	0.07 (0.06)		.24
Watching television	−1.44 (0.25)	<.001	0.49 (0.19)	.01	0.23 (0.05)		<.001
Listening to music or podcasts	0.11 (0.31)	.72	−0.40 (0.24)	.09	0.08 (0.07)		.22
Talking to others	−0.60 (0.36)	.09	0.06 (0.28)	.82	0.09 (0.08)		.24
Texting or checking email	−2.87 (1.06)	.007	−1.70 (0.88)	.05	0.98 (0.23)		<.001
Following content on the internet	−0.19 (1.13)	.87	2.29 (0.88)	.01	0.05 (0.25)		.85
Playing another game	−4.21 (2.13)	.047	0.86 (1.65)	.60	0.60 (0.46)		.20

### Norms

After applying the sampling weights developed for the UAS panel, we computed the norms for the two cognitive tests that were representative of the general US population. Tables 6 and 7 show the weighted averages of test scores by age group with 95% CIs, including participants who reported being distracted.

**Table 6:** Weighted averages of the Stop and Go Switching Task scores with 95% CI by age group (N=5933).

Age group (years)	Baseline average (95% CI)	Reverse baseline average (95% CI)	Nonswitch average (95% CI)	Switch average (95% CI)
18-34	0.80 (0.78-0.82)	0.88 (0.86-0.90)	0.76 (0.75-0.77)	1.25 (1.22-1.29)
35-44	0.90 (0.86-0.93)	0.98 (0.95-1.00)	0.84 (0.82-0.85)	1.36 (1.32-1.40)
45-54	1.04 (1.00-1.07)	1.11 (1.08-1.14)	0.94 (0.92-0.96)	1.55 (1.48-1.62)
55-64	1.29 (1.15-1.42)	1.28 (1.23-1.33)	1.04 (1.02-1.07)	1.61 (1.56-1.67)
65-74	1.44 (1.36-1.52)	1.50 (1.43-1.57)	1.20 (1.16-1.24)	1.86 (1.80-1.92)
≥75	1.50 (1.35-1.64)	1.49 (1.38-1.61)	1.22 (1.16-1.29)	2.01 (1.88-2.14)

**Table 7:** Weighted averages of the Figure Identification test scores with 95% CI by age group (N=6492).

Age group (years)	The Figure Identification test score (95% CI)	Percentage incorrect (95% CI)	Median response time (95% CI)
18-34	47.79 (47.27-48.31)	7.01 (6.64-7.39)	3.56 (3.48-3.64)
35-44	45.01 (44.45-45.56)	6.72 (6.34-7.11)	4.03 (3.93-4.14)
45-54	40.69 (40.10-41.28)	6.80 (6.34-7.25)	4.74 (4.62-4.88)
55-64	38.44 (37.89-38.98)	6.62 (6.19-7.04)	5.20 (5.06-5.34)
65-74	35.52 (35.04-36.00)	6.40 (5.95-6.86)	5.88 (5.73-6.03)
≥75	33.58 (32.80-34.36)	6.53 (5.83-7.23)	6.44 (6.18-6.71)

## Discussion

### Principal Findings

Surveys are increasingly being administered over the internet, posing questions about the quality of web-based information. This is especially true for measures of cognition. Cognitive tests have traditionally been administered in controlled environments under the supervision of a trained psychometrist, whereas administration in web surveys is potentially subject to spurious differences related to the type of device used by the respondent and distractions outside the control of the survey agency [13,16,40]. Nevertheless, assessing participants’ cognitive abilities in large, nationally representative samples is often desirable [7,8]. In this paper, we studied web-based versions of 2 types of speeded cognitive tests—a switching test (SGST) to measure executive functioning and a matching test (Figure Identification test) to measure perceptual speed—in a nationally representative sample of US adults.

We developed the tests and their implementation iteratively through pilot tests and feedback from the participants in those pilots. Importantly, we imposed no restrictions on the system or hardware requirements, with the goal of broadly accommodating all devices that participants might have available. The final versions worked well for a large majority of participants in the full sample, and most participants reported experiencing no difficulties seeing the text and buttons clearly or navigating the screens. Somewhat greater difficulties with the self-administered tests were reported among the smaller subsets of participants who either did not finish the tests or provided <70% accurate answers and were, therefore, not scored. It is also noteworthy that the rate of participants who did not meet this accuracy criterion was about twice as high (910/7039, 12.93%) for the SGST administered in this study compared with a previous report of telephone-administered SGST (262/4268, 6.1%) [7]. Further investigation revealed that 36.4% (332/912) of the excluded SGST sample failed in almost all the reverse baseline trials. This group’s accuracy rate was high for the normal baseline trials as well as the practice trials of the reverse baseline condition. They also performed reasonably well in trials alternating between normal and reverse conditions (ie, switch and nonswitch trials), which suggests that these respondents might have mistakenly applied the normal baseline rules to the reverse baseline trials. For future respondents, we further modified the instructions by reiterating the reverse baseline rules between the practice and scorable trials of the same condition. As the development of web-based cognitive tests in the UAS is an ongoing process, further reduction of the remaining technical difficulties could continue to optimize test administration to ensure that the tests work as intended for all participants.

To compare any device effects, we asked individuals who had both a keyboard-based device and a touch screen–based device to perform the tests once on each device, for which we randomized the order. Successfully implementing this experimental study design component ultimately proved challenging as participants did not own both devices, did not agree to complete the tests on both devices, or did not use both devices as instructed, which resulted in a much reduced and nonrepresentative sample. This highlights the challenges frequently associated with executing randomized experiments in the context of large-scale internet panels [41]. Nevertheless, when comparing participants’ scores for the first session, which yielded a very high participation rate, we found that keyboard- and touch screen-based devices yielded very similar scores in terms of participants’ average cognitive performance. This was corroborated by the results from within-person analyses in the subsample of participants who successfully completed the experimental study design.

We found that older age was associated with worse scores on both cognitive tests, regardless of the device type. The observed worsening of scores was evident over the full adult age range, consistent with the theoretically expected age-normative cognitive trajectories [42]. Although we cannot rule out that the relationship with age is partially because of differences in familiarity with digital devices, the correlations between age and cognitive scores were consistent in magnitude with those previously reported for the original tests administered with traditional assessment formats. Scores on the SGST have been reported to correlate with age at 0.34 when the test was administered via telephone in the Midlife in the United States study [7], consistent with the correlations (ranging from 0.32 to 0.50) observed in this study. Similarly, age correlations for the traditional paper-and-pencil administered Figure Identification test ranged from −0.46 to −0.55 across waves in the Swedish Adoption/Twin Study of Aging [23,43], comparable in magnitude to those in this study (range −0.49 to −0.61). Furthermore, the tests themselves require only minimal familiarity with digital devices (pressing a specific key or button), which UAS members likely had already acquired in previous surveys in which they participated.

To date, only a few studies have examined the influence of different test settings on cognitive test scores [12,44]. To study the potential effects of the lack of a controlled environment, we asked the participants about their location and the number of potential distractions in day-to-day life during the testing. Although participants were allowed to complete the tests in any location, the vast majority completed them in their home environment, in part because data collection took place during the COVID-19 pandemic between November 2020 and April 2021. This may have reduced environmental influences to some extent. Nevertheless, a nontrivial number of participants were interrupted during the test or engaged in simultaneous activities that could be distracting, especially watching television or listening to music. Furthermore, our regression analyses showed that many of these environmental factors significantly affected the cognitive test scores. To evaluate the magnitude of these effects, apart from their statistical significance, it is useful to view them in the context of the corresponding age effects on cognitive scores. For the Figure Identification test, being interrupted was associated with a reduction of 0.89 figures correctly solved within the time limit (Table 5), which is approximately the same amount as the reduction in the Figure Identification test scores that would be expected for 3 years increase in age (Table 7). Similarly, for the SGST, being interrupted reduced participants’ performance by approximately 0.08 to 0.15 seconds on average (Table 4), which corresponds with a performance reduction that would be expected for approximately 4 to 9 years increase in age (Table 6). This suggests that environmental distractions may have a nontrivial yet modest biasing impact when using observed cognitive scores in population-based research.

These environmental distractions occurred despite our instructing respondents to set aside uninterrupted time to complete the cognitive tasks. We developed an expanded warning about potential interruptions that concludes by requiring the participant to respond affirmatively that now is a good time to complete the tasks. We recommend that this approach be incorporated by other researchers using remote testing.

Our study had several limitations that should be considered. First, a nontrivial number of UAS panelists were not scored as they did not meet the accuracy threshold. Further investigation is important to understand the reasons (eg, to what extent this was because of inattentiveness vs the participants’ lack of capability to complete the tasks) and the extent to which this introduced systematic bias in this study. Second, we developed tests in a probability panel, which mitigates the digital divide known to be associated with socioeconomic status by providing internet-connected devices to those who need them. However, it is possible that participants with low computer skills and poorer cognitive functioning were less likely to participate in this study. Third, our sample was predominantly English-speaking; the very small proportion of Spanish-speaking participants did not allow meaningful analyses or comparisons across language subgroups. Although the cognitive tests studied in this paper are less language dependent than many other commonly used neurocognitive tests, caution should be taken when generalizing the study findings to specific subpopulations. Fourth, although the comparison of scores across device types was the primary objective of this study, there are many fine-grained differences within each of the device types (eg, screen or display size, keyboard, and touch screen functionality) that we did not examine, which could affect cognitive test scores. Fifth, although our sample included a group of older adults aged ≥75 years, the sample sizes were relatively small (311/6129, 5.07% for SGST; 419/6717, 6.24% for the Figure Identification test). Findings specific to this age group, such as the norms on cognitive scores, should be validated in larger samples in the future. Finally, our results suggest a small but unignorable impact of environmental distractions on test performance, which is an inherent problem for self-administered tests in general. In part, such inattentiveness may also lead to some participants failing to meet the accuracy threshold. Future studies are indispensable to detangle inattention from incapacity and explore ways of improving attention and reducing distractions.

### Conclusions

Keeping these caveats in mind, we conclude that our rigorously developed cognitive measures are not unduly biased by the relative lack of standardization associated with web-based cognitive testing environments. The degree of error introduced by variations in devices and environments does not undermine the sensitivity of the measures used to detect group differences for research purposes. At the same time, we caution that the errors may be substantial enough to impede the accuracy of clinical decisions for individuals.

Our normative data, as presented, are suitable for interpreting SGST and the Figure Identification test results from future studies of English-speaking speaking US adult populations. To date, very few studies have provided normative data for web-based self-administered cognitive tests, and the quality of the norms provided here benefits from sampling weights developed within an existing probability-based sample and from larger sample sizes compared with previously reported web-based cognitive test data [1,3,4,12]. However, we also note that the samples used here are smaller than those used for validating and norming psychological tests in other areas such as quality of life research [45]; therefore, these numbers should be used with caution until more experience with these tests in web surveys has been gained.

## Appendix

Multimedia Appendix 1 Demographic characteristics of the participants who completed the tests on both device types and met 70% correctness (included in the within-person comparison of test scores) and who owned both device types but did not meet these criteria (excluded from the within-person comparison).

Multimedia Appendix 2 Within-person comparison of cognitive scores across device types.

## Abbreviations

SGST: Stop and Go Switching Task
UAS: Understanding America Study
