# Supplementary material for: Self-administered Web-Based Tests of Executive Functioning and Perceptual Speed: Measurement Development Study With a Large Probability-Based Survey Panel
Source: J Med Internet Res. 2022 May 9;24(5):e34347. doi: 10.2196/34347 (PMC9127643; doi:10.2196/34347)
Supplement: Multimedia Appendix 1 [file jmir_v24i5e34347_app1.docx]

| Participant | | Stop and Go Switching Task | | | Figure Identification test | | |
| --- | --- | --- | --- | --- | --- | --- | --- |
| Characteristics | | Included | Excluded | *P* | Included | Excluded | *P* |
|  |  | (N=1770), n (%) | (N=2041), n (%) |  | (N=1892), n (%) | (N=1501), n (%) |  |
|  |  |  |  |  |  |  |  |
| **Age (years)** | |  |  | <.001 |  |  | <.001 |
|  | 18-34 | 408 (23.05) | 394 (19.30) |  | 403 (21.30) | 309 (20.59) |  |
|  | 35-44 | 427 (24.12) | 367 (17.98) |  | 442 (23.36) | 264 (17.59) |  |
|  | 45-54 | 362 (20.45) | 354 (17.34) |  | 375 (19.82) | 266 (17.72) |  |
|  | 55-64 | 304 (17.18) | 402 (19.70) |  | 349 (18.45) | 301 (20.05) |  |
|  | 65-74 | 219 (12.37) | 381 (18.67) |  | 256 (13.53) | 261 (17.39) |  |
|  | ≥75 | 46 (2.60) | 141 (6.91) |  | 67 (3.54) | 100 (6.66) |  |
| **Race** | |  |  | <.001 |  |  | <.001 |
|  | Non-Hispanic White | 1238 (69.94) | 1234 (60.46) |  | 1312 (69.34) | 886 (59.03) |  |
|  | Non-Hispanic Black | 93 (5.25) | 196 (9.60) |  | 111 (5.87) | 136 (9.1) |  |
|  | Hispanic | 232 (13.11) | 380 (18.62) |  | 251 (13.27) | 281 (18.8) |  |
|  | Non-Hispanic other | 204 (11.53) | 228 (11.17) |  | 217 (11.47) | 193 (12.9) |  |
| **Sex** | |  |  | .047 |  |  | .211 |
|  | Men | 749 (42.32) | 799 (39.15) |  | 775 (40.96) | 583 (38.84) |  |
|  | Women | 1021 (57.68) | 1242 (60.85) |  | 1117 (59.04) | 918 (61.16) |  |
| **Education** | |  |  | <.001 |  |  | <.001 |
|  | High school or less | 179 (10.11) | 398 (19.50) |  | 206 (10.89) | 292 (19.45) |  |
|  | Some college | 516 (29.15) | 762 (37.33) |  | 574 (30.34) | 564 (37.57) |  |
|  | Bachelor or more | 1075 (60.73) | 880 (43.12) |  | 1112 (58.77) | 645 (42.97) |  |
| **Household income (US $)** | |  |  | <.001 |  |  | <.001 |
|  | ≤24,999 | 170 (9.60) | 360 (17.64) |  | 196 (10.36) | 279 (18.59) |  |
|  | 25,000-49,999 | 283 (15.99) | 434 (21.26) |  | 309 (16.33) | 306 (20.39) |  |
|  | 50,000-99,999 | 642 (36.27) | 673 (32.97) |  | 685 (36.21) | 489 (32.58) |  |
|  | ≥100,000 | 671 (37.91) | 564 (27.63) |  | 698 (36.89) | 422 (28.11) |  |
