# Supplementary material for: Self-administered Web-Based Tests of Executive Functioning and Perceptual Speed: Measurement Development Study With a Large Probability-Based Survey Panel
Source: J Med Internet Res. 2022 May 9;24(5):e34347. doi: 10.2196/34347 (PMC9127643; doi:10.2196/34347)
Supplement: Multimedia Appendix 2 [file jmir_v24i5e34347_app2.docx]

| Cognitive scores | | Values, mean (SD) | | Cohen *d* (95% CI) | Correlation across device types |
| --- | --- | --- | --- | --- | --- |
|  |  | Keyboard | Touchscreen |  |  |
| **Stop and Go Switching Task^a^** | |  |  |  |  |
|  | Baseline^b^ | 0.87 (0.34) | 0.98 (0.44) | 0.26 (0.19, 0.33) | 0.37 |
|  | Reverse Baseline^b^ | 0.94 (0.34) | 1.08 (0.47) | 0.34 (0.28, 0.41) | 0.45 |
|  | Nonswitch^a^ | 0.81 (0.20) | 0.90 (0.30) | 0.35 (0.29, 0.42) | 0.66 |
|  | Switch^a^ | 1.30 (0.50) | 1.37 (0.51) | 0.14 (0.08, 0.21) | 0.53 |
| **Figure Identification test^c^** | |  |  |  |  |
|  | FigID score^d^ | 46.15 (8.43) | 45.83 (8.59) | -0.04 (-0.01, -0.07) | 0.77 |
|  | Percent figures incorrect^e^ | 4.69 (4.16) | 5.73 (4.76) | 0.23 (0.19, 0.28) | 0.57 |
|  | Median response times^b^ | 4.03 (1.45) | 3.99 (1.48) | -0.03 (-0.05, 0.001) | 0.83 |

^a^For Stop and Go Switching Task, n=1770.

^b^Means and SDs are presented for seconds.

^c^For the Figure Identification test, n=1892.

^d^Means and SDs are presented for the number of figures.

^e^Means and SDs are presented for percentage.
